# Evaluating the Usefulness and Ease of Use of a Next-Generation–Connected Drug Delivery Device for Growth Hormone Therapy: Qualitative Study of Health Care Professionals’ Perceptions

**DOI:** 10.2196/46893

**Published:** 2023-08-02

**Authors:** José I Labarta, Paul Dimitri, Matthew Keiser, Ekaterina Koledova, Octavio Rivera-Romero

**Affiliations:** 1 Unit of Endocrinology Department of Pediatrics Hospital Universitario Miguel Servet Zaragoza Spain; 2 Instituto de Investigación Sanitaria Aragón Zaragoza Spain; 3 Department of Paediatric Endocrinology Sheffield Children's NHS Foundation Trust Sheffield United Kingdom; 4 Ares Trading SA (an affiliate of Merck KGaA) Eysins Switzerland; 5 Global Medical Affairs Cardiometabolic & Endocrinology Merck Healthcare KGaA Darmstadt Germany; 6 Electronic Technology Department Universidad de Sevilla Seville Spain

**Keywords:** connected health, growth hormone deficiency, participatory health informatics, recombinant human growth hormone, technology acceptance, mobile phone

## Abstract

**Background:**

Digital solutions targeting children’s health have become an increasingly important element in the provision of integrated health care. For the treatment of growth hormone deficiency (GHD), a unique connected device is available to facilitate the delivery of recombinant human growth hormone (r-hGH) by automating the daily injection process and collecting injection data such that accurate adherence information is available to health care professionals (HCPs), caregivers, and patients. The adoption of such digital solutions requires a good understanding of the perspectives of HCPs as key stakeholders because they leverage data collection and prescribe these solutions to their patients.

**Objective:**

This study aimed to evaluate the third generation of the easypod device (EP3) for the delivery of r-hGH treatment from the HCP perspective, with a focus on perceived usefulness and ease of use.

**Methods:**

A qualitative study was conducted, based on a participatory workshop conducted in Zaragoza, Spain, with 10 HCPs experienced in the management of pediatric GHD from 7 reference hospitals in Spain. Several activities were designed to promote discussion among participants about predefined topics based on the Technology Acceptance Model and the Unified Theory of Acceptance and Use of Technology to provide their perceptions about the new device.

**Results:**

Participants reported 2 key advantages of EP3 over previous easypod generations: the touch screen interface and the real-time data transmission functionality. All participants (10/10, 100%) agreed that the new device should be part of a digital health ecosystem that provides complementary functionalities including data analysis.

**Conclusions:**

This study explored the perceived value of the EP3 autoinjector device for the treatment of GHD by HCPs. HCPs rated the new capabilities of the device as having substantial improvements and concluded that it was highly recommendable for clinical practice. EP3 will enhance decision-making and allow for more personalized care of patients receiving r-hGH.

## Introduction

### Background

Digital solutions targeting children’s health outcomes are rapidly gaining traction in health care [1]. The World Health Organization introduced *digital health* as a broad umbrella term encompassing eHealth, which refers to the design and use of information and communication technologies to support the promotion, prevention, treatment, and maintenance of health outcomes [2]. With the introduction and implementation of new digital health solutions, there needs to be an understanding of how these technologies can best be implemented within clinical care pathways; in the patient’s home; and the broader environment in a way that supports connectivity locally, regionally, and globally. eHealth includes mobile health (mHealth)—health-related services delivered via mobile communications devices [3]—which allows health care services to be accessed and delivered remotely in real-world settings. This enables more accurate real-time collection of a large amount of data about health conditions and behaviors [4-6], using advanced analytical techniques to assess, for example, adherence and the effects of treatment on clinical outcomes; these data can be collated at an individual or population-based level. Communication, education, social participation, and treatment reminders are other examples of how mobile-enabled health care services can be used. Such technological developments are triggering a paradigm shift from standard face-to-face interventions toward a more integrated, patient-centered, personalized, and potentially more cost-effective health care approach. mHealth has the potential to improve treatment outcomes and patients’ quality of life [7], as shown by the use of SMS text message reminders to improve medication adherence and perceived quality of life in adolescents with asthma [8] and digitally enabled continuous glucose monitoring in children and adolescents with type 1 diabetes mellitus [9].

### Developing Digital Health Tools

Designing digital health tools for children and adolescents requires specific considerations that relate to the anatomical, physiological, and psychosocial changes during their development [10]. These include changes in children’s developmental characteristics as they mature, parent-child dynamics, and the transition of children into adult health care [11]. Type 1 diabetes mellitus is a good example of where it is particularly important to effectively manage the transition from pediatric to adult health care [12], during which technologies can play a fundamental role [9,13,14]. An approach to developing digital tools to support pediatric health care is to integrate user-centered design (UCD). UCD is an evidence-based framework informed by the needs and understanding of specific user groups at every stage of the design process and is invaluable in the development of mHealth technologies [15,16]. It is part of the International Organization for Standardization (ISO) standard “Ergonomics of human-system interaction—Part 210: Human-centered design for interactive systems” [17] and is endorsed by the World Health Organization [18]. UCD aims to create solutions that meet the specific needs, characteristics, preferences, and tasks of the intended users [17,19]. Systems developed using an iterative design process following UCD principles are easy to use and learn, reach high user acceptance and satisfaction levels, and reduce the number of user errors [17,19,20]. Most UCD methods in health care involve service users and service providers in the different stages of the development process [19-21], and involving health care professionals (HCPs) in the development of such solutions may have a positive impact on their perceived reliability [22,23]. Despite the apparent value of UCD, a systematic review of 69 randomized studies of mobile apps designed to support patients with chronic diseases reported that robust developmental factors are rarely adopted during the design stage, with approximately only one-third of the studies reporting user or HCP involvement [24]. Examples of where UCD was applied to the development of digital health solutions for pediatric health care that did involve HCPs include the mHealth tool, the Pictorial Support in Person-Centered Care for Children app, and the development of an electronic cross-facility health record for pediatric palliative care [25,26].

### Digital Health Tools for Growth Hormone Deficiency

Digital health tools have been used to support patients in the self-management of pediatric endocrine disorders, such as growth hormone deficiency (GHD). Long-term management of GHD is often challenging for children, their caregivers, and HCPs, as treatment requires daily injections over many years, either self-administered or administered by caregivers [27]. Connected medical devices can be used to facilitate this process by automating the injection, delivering accurate predetermined doses, improving comfort, and reducing injection-related anxiety. Using these devices to monitor therapy by digitally recording daily injections can improve adherence to such long-term therapy through the early detection of suboptimal adherence and, therefore, appropriate intervention by HCPs. Poor adherence can lead to reduced efficacy, increased comorbidities, and increased health care costs and has long been associated with growth hormone (GH) treatment and thus underpins the need and value of objectively measuring adherence by a connected device to drive early intervention [28]. Currently, there is only 1 digitally enhanced, connected autoinjector available to deliver recombinant human GH (r-hGH; somatropin [Saizen], the health care business of Merck KGaA, Darmstadt, Germany) treatment—the easypod (the health care business of Merck KGaA, Darmstadt, Germany) device, which has, so far, been approved in >40 countries. This device has been widely used in pediatric research and practice to improve treatment adherence [29] by facilitating the collection of real-time injection data, so that reliable, accurate information about adherence to treatment is available to HCPs for assessment. Furthermore, population data from these devices provide a means of developing prediction tools to support clinical decision-making [30]. As users and prescribers of new digital health technologies to support pediatric growth therapies, it is important to garner HCPs’ perspectives about the acceptance of these devices during their design and development to test usability, feasibility, and acceptability; this was the rationale for conducting this study. Several qualitative studies exploring HCPs’ perceptions about factors and barriers related to digital health acceptance in endocrinology and other therapy areas have been published in the scientific literature [29,31-34]. Some have explored HCPs’ perceptions about mHealth tools used in children’s health care [29,31-37], concluding that early engagement of end users is critical to the development and effective implementation of such tools to enhance patient-centric care. Notably, a mixed methods formative research study has explored technology acceptance and the use of digital health tools for the emotional support of parents of children undergoing GH treatment, using educational content to help parents and caregivers understand their children’s treatment journey [38]. However, to the best of our knowledge, no study has explored HCPs’ perceptions about the acceptance of mHealth solutions (and their technological evolution) to support pediatric GH therapy. For example, digital interventions based on recorded adherence data have been implemented in the context of r-hGH treatment [39].

The third generation of the easypod device (EP3) was designed with patients and caregivers in mind; however, evaluations using UCD methods to better understand the HCPs’ perspective to support the implementation and acceptance of the device in relation to their specific needs (eg, by better understanding the barriers to implementation and advantages of the device) were not performed during the development phase.

### Objectives

Therefore, to add to the existing published literature and to build upon the insights from previous studies, this study was conducted to assess 2 constructs of technology acceptance of EP3—*perceived usefulness* and *ease of use*—compared with the current digital health device used to support and deliver pediatric r-hGH therapy from an HCP perspective.

## Methods

### Methodological Models

Several Technology Acceptance Models (TAMs) and theories have been developed to explain the intention to use technological solutions [40-47]. As an example, the TAM is a behavioral model of user acceptance of technology that has been widely used in research [40]. It posits that the perceived usefulness and perceived ease of use of a digital solution predict the intention to use it and, therefore, its actual use. Several versions of TAM have been developed incorporating additional factors such as social norms [43,44]. As this study aims to explore how the technological advances could have an impact on HCP perspectives, only the core factors that are directly related to the technology being assessed have been considered (ie, perceived usefulness and ease of use). In this regard, perceived usefulness is defined as “the degree to which a person believes that using a particular system would enhance his or her job performance,” whereas perceived ease of use is defined as “the degree to which a person believes that using a particular system would be free of effort.”

The Unified Theory of Acceptance and Use of Technology (UTAUT) [45] identifies 4 main constructs that play a significant role as direct determinants of user acceptance and use behavior: performance expectancy, effort expectancy, social influence, and facilitating conditions. The first 2 are related to the abovementioned TAM’s constructs. Performance expectancy is defined as “the degree to which an individual believes that using the system will help him or her to attain gains in performance.” According to UTAUT, this construct is the strongest predictor of intent to use. It is directly related to perceived usefulness defined in the TAM. Effort expectancy is defined as “the degree of ease associated with the use of the system” and encapsulates the same concept as the TAM’s perceived ease of use construct.

In the health domain, Kim and Park [42] developed the Health Information TAM (HITAM). This model expands upon the TAM by adding specific factors related to health. Perceived usefulness and perceived ease of use are still considered as significant mediators of user’s attitude, which directly influences the behavioral intention and, hence, use. An additional core construct is also included in HITAM, namely, perceived threat, which is derived from the Health Belief Model (HBM) [48]. The HBM is a social cognition model used to explain health behavior change. It suggests that belief in a personal threat, together with belief in the effectiveness of the proposed behavior, predicts the likelihood of engaging in that behavior.

Finally, Wang et al [47] defined a model that integrates UTAUT and Task-Technology Fit (TTF) [49] to understand how consumers accept health care wearable devices. TTF posits that “for a technology to have a positive impact on individual performance, the technology: (1) must be utilised and (2) must be a good fit with the tasks it supports” [49]. This model incorporates components derived from TTF (technology characteristics, task characteristics, and TTF) to UTAUT. Wang et al [47] found that performance expectancy was the most important determinant of behavioral intention. They also determined that technology characteristics could positively predict effort expectancy, whereas TTF directly influenced behavioral intention through the mediating role of performance expectancy.

In this study, we focused on 2 constructs included in the TAM—*perceived usefulness* and *perceived ease of use—*which are also components of the UTAUT, HITAM, and HBM. TAM and UTAUT are general acceptance models and could be used as the basis for studies in any domain; however, HITAM and HBM are models specifically defined for the health domain. Therefore, as our study focused on these 2 main constructs, the theoretical foundations from both general acceptance and health-related models were valid to explore how pediatric HCPs perceive the potential impact of technology evolution on the acceptance of an mHealth device, namely, easypod.

### Study Design

**Figure 1:**
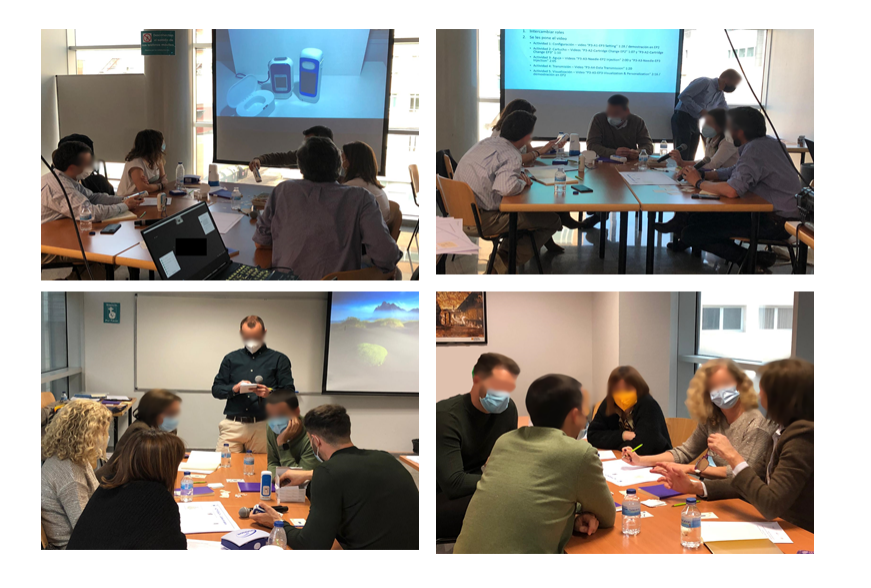
Photos of participants taken during the workshop sessions.

This theory-driven qualitative study was conducted through a participatory workshop involving 10 HCPs (n=6, 60% endocrinologists; n=2, 20% nurses; and n=2, 20% pharmacists), with the workshop lasting 3 hours (Figure 1). Several predefined questions designed based on *perceived usefulness* and *perceived ease of use* were discussed during the workshop session.

### Study Cases

**Figure 2:**
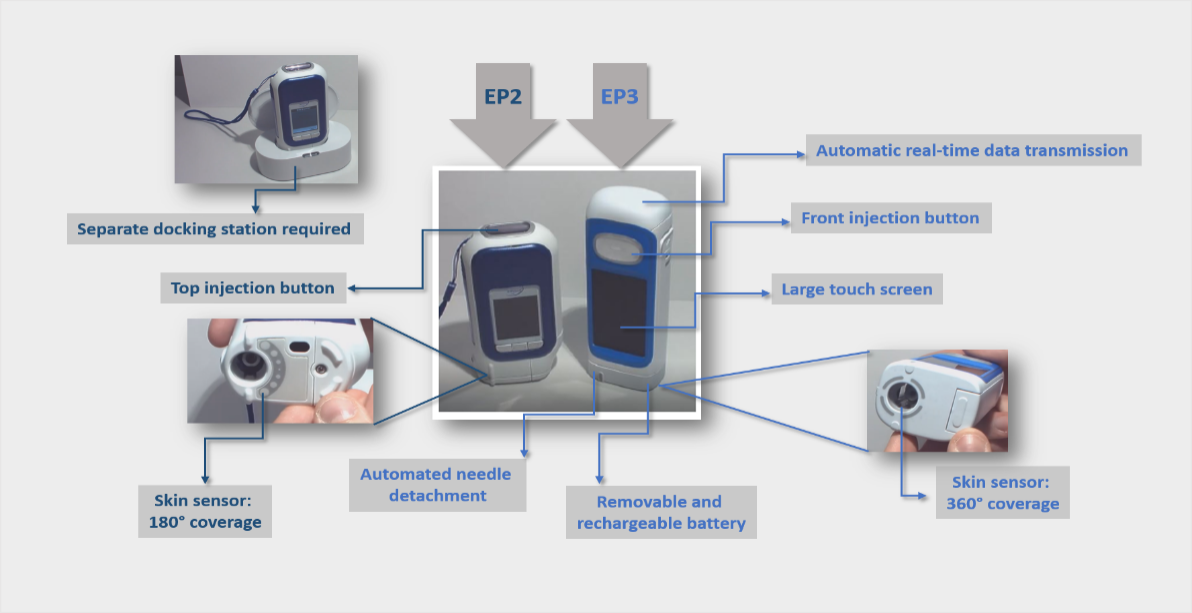
Attributes of the current easypod device (EP2) and the third generation of the easypod device (EP3).

Easypod is the only digitally enhanced, connected autoinjector device available to deliver r-hGH treatment. Therefore, we used 2 versions of this device as the comparable study cases: the current easypod device (EP2) and EP3 that is currently in development to deliver r-hGH and monitor real-time adherence to therapy (Figure 2).

Both devices record the date, time, and dose received, but EP2 cannot transmit these data until the user or carer places it on a separate docking station and activates transmission. In contrast, EP3 transmits the data automatically, with no requirement for user activation or a separate docking station for data transmission.

EP3 is taller and slimmer than EP2 and has a removable and rechargeable battery; a large, easier-to-read touch screen; and a skin sensor with 360° coverage, enabling improved skin contact compared with the 180° coverage with EP2; thus, it is intended to make injections easier and more accurate. The injection button on EP3 is at the front of the device, whereas on EP2, it is at the top. The needle is hidden on both devices to minimize needle phobia and patient anxiety, with the additional feature of automated needle detachment on EP3. The comfort settings (injection speed; injection depth; and needle speed, which can be adjusted by an HCP according to patient preference; and injection time, ie, the duration for which the needle remains in the skin) are a feature shared by the 2 devices (Figure 2).

Regarding safety, EP3 will comply with all the latest and relevant standards for medical devices (ISO 11608, International Electrotechnical Commission 60601, and ISO 62304).

### Study Setting

The participatory workshop, upon which this qualitative study was based, was conducted at the University Hospital Miguel Servet, Zaragoza, Spain, on February 23, 2022, in 2 meeting rooms situated in proximity on the same floor of the hospital. Both rooms had the necessary technical equipment (video projector, audio system, and computer) required for the sessions. The workshop was conducted in Spain, as there is a representative sample of physicians who have used digital health solutions available in clinical practice there.

### Study Participants

Participants included HCPs from 7 different hospitals from 6 different regions in Spain, with experience in the management of growth disorders using r-hGH treatment in pediatric patients, either with or without previous experience of using EP2. The management of GH therapy in Spain involves multidisciplinary teams comprising pediatric endocrinologists, nurses, and pharmacists. As such, we sought views from representatives of these disciplines about the usefulness and ease of use of EP2 and EP3, ensuring that the sex and expertise of participants (endocrinologist, nurse, or pharmacist) were considered when selecting the final sample of participants. Participants were grouped into 2 teams, balanced in terms of their professional expertise and sex, and each team was led by a facilitator. Both facilitators had experience of conducting qualitative studies.

### Ethics Approval and Informed Consent

Ethics approval for this study was obtained from the ethics committee of research of the Universidad de Sevilla (ID 2593-N-21). Participants’ agreement for participation was obtained through an informed consent process.

### Participatory Workshop

#### Workshop Design

**Figure 3:**
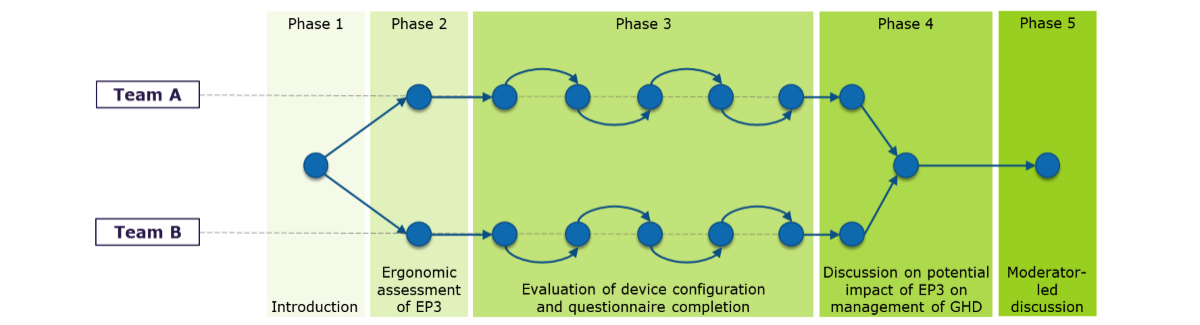
Phases of the workshop.

The participatory workshop consisted of several activities in which participants discussed a set of predefined topics. The workshop was designed by 2 experts in participatory health informatics. Several procedures and materials were designed to create an appropriate working environment to facilitate discussions. The topics to be discussed incorporated the 2 constructs of the TAM (*perceived usefulness* and *perceived ease of use*) and were led by a multidisciplinary team including experts in digital health, participatory health informatics, and technology acceptance and HCPs. Before beginning each activity, the facilitator provided clear instructions to participants about how to perform the activity, and any questions were resolved. These activities were grouped into 5 phases (Figure 3).

#### Phase 1

*Phase 1* aimed to briefly introduce the project; its objectives, facilitators, and phases; and specific tasks to be performed during the workshop. All participants were in the same room and the facilitator asked them to briefly introduce themselves. We also clarified technical terms such as “digital health solution” or “device,” and any questions were resolved. Finally, participants were grouped into 2 teams, and each team performed the activities in a separate room.

#### Phase 2

*Phase 2 c*onsisted of 2 activities (activities 1 and 2) that were performed independently by each team. During activity 1, participants discussed the ergonomics of the packaging (the cases in which the devices are held and transported) of each device (EP2 and EP3). The facilitators provided participants with the packaging for each device. Participants spent a short time inspecting the cases, trying to open and close them, and compared their physical characteristics. Any questions were resolved. The facilitator then asked participants to highlight the strengths and weaknesses of the packaging for the 2 devices, considering 2 scenarios: from the user and the caregiver perspectives.

**Figure 4:**
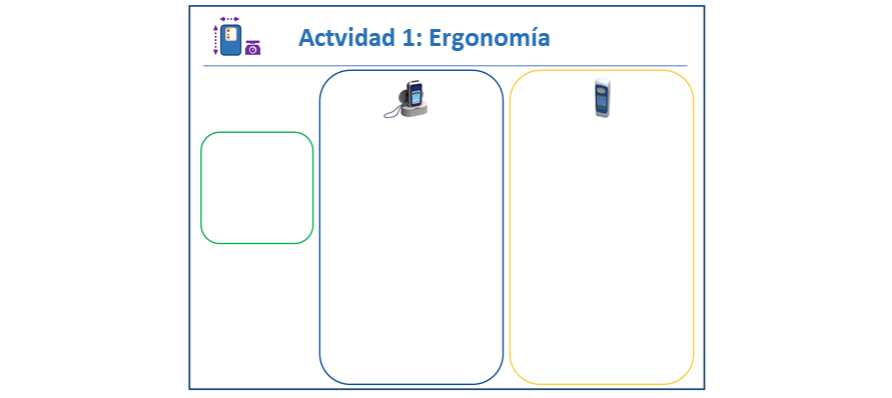
An example of one of the sticky cards representing the topics predefined for activity 2.

Activity 2 focused on the ergonomics of the 2 devices. At the beginning of this activity, the facilitators provided participants with a prototype of each device, the dimensions and weights of which were equal to the commercial devices but did not implement all their software functionalities. Participants spent a short time inspecting the devices, performed a simulated injection, and compared their physical characteristics. In addition, an introductory video presenting the main characteristics of each device was played, and any questions were resolved. The facilitators then provided the teams with an activity 3 template to prompt discussions and a set of sticky cards that represented the topics predefined for this activity (an example of which is shown in Figure 4). Each sticky card comprised short text and imagery representing a predefined topic. Next, participants randomly selected a topic card and stuck it on to the template. Participants discussed the selected topic and highlighted the strengths and weaknesses of both devices. A participant summarized the opinions reported by the team, making brief notes on the template. Once the discussions were completed, a new topic was randomly selected, and the same procedure was repeated until all predefined topics were discussed or the time to complete the activity was reached. Some examples of the predefined topics for this activity were the appropriateness of device weight, dimensions, screen location, administration button size, administration button location, and feedback light location.

#### Phase 3

The objective of *phase 3* was to explore participants’ opinions about the differences and similarities of both devices when they are used to manage treatments and the potential impact on the technology acceptance perceived by participants. The complete process to perform treatment administration using the easypod devices was split into 6 main tasks: dose configuration, cartridge replacement, needle detachment and attachment, performing injections, data transmission, and providing user feedback. We designed an activity for each task (activities 3-7) that was independently performed by each team. An activity 3 template and a set of sticky cards representing the predefined topics were designed for each activity. The procedures followed for each activity were the same as those used in activity 2. First, a short video explaining how to perform the corresponding task using both devices was played, and any questions were resolved. Next, a topic was randomly selected and discussed among participants. Participants’ opinions were noted down on the template during discussions. The process of selecting a topic and discussing it was repeated until the time to complete the activity was reached or until all predefined topics were discussed. Some examples of the predefined topics for these activities were perceived ease of performing the task, perceived safety while performing the task, potential human errors, perceived ease of interaction with the device, perceived ease of teaching the task to HCPs, patients, and caregivers, perceived effort required to perform the task, and potential benefits of using the device.

#### Phase 4

*Phase 4* began with an individual activity (activity 8). In this activity, each participant individually completed a questionnaire. The main idea of this individual activity was to give participants the opportunity to report any opinion that they did not provide during the previous activities. The questionnaire consisted of 25 items aimed to assess the participants’ perceptions about how the improvements included in EP3 influenced acceptance in terms of 4 domains: *perceived usefulness, perceived ease of use, ease of learning,* and *intention to use and recommend*. Each item of the questionnaire consisted of 2 components: a 5-point Likert question and an open-ended question. The first component aimed to present the specific question to be discussed and to allow participants to think about its perceived relevance. The second component asked participants to justify the score assigned. Although a quantitative questionnaire was used, we analyzed the data collected in this activity using a qualitative approach. The list of items included in the questionnaire is presented in Multimedia Appendix 1, the results of which were used in a descriptive manner, with the aim of providing new insights and observations that were not otherwise reported during the workshop; mean Likert scores according to domain are presented in Multimedia Appendix 1.

Next, the 2 groups of participants were brought together to present their findings about the most relevant issues reported during the previous activities, potential impact of the improvements included in EP3 on the management of r-hGH treatment, and role of these advances in the broader digital ecosystem. During this phase, we sought to present the perceptions of both teams and encouraged the participants from each team to discuss their opinions in depth. Facilitators summarized the most relevant comments reported by participants during the previous activities and then presented these relevant issues separately to prompt discussion among the participants.

#### Phase 5

Finally, during *phase 5*, some statements representing the most relevant findings were presented to all participants, and facilitators asked the participants to validate these conclusions and gave them the opportunity to add some additional comments to clarify them.

### Data Analysis

The workshop session was audio recorded. The audio recordings were reviewed by a researcher, after which, relevant comments were transcribed, and information from the facilitators’ notes and text included in the templates were combined into the study data set. Owing to the small sample size of participants, we did not seek to determine the statistical significance or generalizability of the quantitative data collected using the defined questionnaire but, instead, to describe their opinions. In this regard, OR-R reviewed the scores assigned by participants to questions included in the questionnaire and checked whether any additional opinions were provided, to ensure that they were of a qualitative nature. These additional opinions were included in the data set. Then, the data collected in this study were explored qualitatively using an inductive approach following a simplified theory-guided thematic analysis for qualitative data [50]. OR-R reviewed all collected data, coded them, and defined themes, after which, all authors reviewed the proposed themes and refined them until consensus was reached. For this study, the quantitative data were not analyzed.

## Results

### Overview

Teams were created from the participating HCPs, considering their professional backgrounds (3/10, 30% endocrinologists; 1/10, 10% nurses; and 1/10, 10% pharmacists in each team). Overall, 5 themes were identified: simplified touch screen interface, real-time data transmission, administration safety, digital ecosystem, and additional improvements (Table 1). All scores reflected conclusions that aligned with the findings from the qualitative data (Multimedia Appendix 1). As an example of the perceived impact of the technological evolution of EP3, HCPs perceived the improvements as having a positive impact on its usefulness and ease of use. These quantitative data showed a high predisposition of the HCPs to use and recommend the new-generation device, demonstrating that they perceived it as an important advancement.

**Table 1:** The 5 themes identified during the workshop.

Themes	Participants’ insights (verbatim)
Simplified touch screen interface	“The touch screen is very clear and visible.” “More intuitive because of the touch screen.” “Bigger screen, better resolution, and touch screen.” “Text is easier to read.” “Children are more familiar with the use of touch screen.” “Bigger screen size makes easy the configuration.” “Provides more information in the screen.” “The administration button in the frontal location could result in unintended interactions with the touch screen.”^a^ “New device could cause errors because of the location of the touch screen close to the button.”^a^ “User could touch accidentally the screen while he/she is administrating his/her treatment.”^a^
Real-time data transmission	“It is the main improvement, and it is a big advance.” “It is a crucial component.” “The adherence data transmission is a key factor.” “Avoid patients/caregivers to forget completing the process.” “Real-time data.” “Patients/caregivers have one less action to do.” “Independency of family will/skills.” “Better adherence monitoring, especially in non-adherent patients.” “Automatic data transmission will improve the control of adherence for patient, caregiver and HCP^b^.” “Actions from patients/caregivers are not required.” “Automatic transmission does not require any action by users.” “Data transmission is independent from users.” “Patients/caregivers are aware HCPs are accessing data in real-time, therefore this fact will impact positively on their behaviours.”
Administration safety	“EP3^c^ allows a safety process but EP2^d^ did it [similar EP3 and EP2].” “More intuitive.” “EP3 is very easy to use.” “Clinical settings must use a more secure access technique.”
Digital ecosystem	“Real adherence data allows to make better decisions.” “Improved data usage but care will be the same.”
Additional improvements	“Removing the needle de-attachment button is a big advantage.” “Especially because of the simpler needle de-attachment process.” “Especially, EP3 minimises problems because of the needle de-attachment process.” “Easier to use and more sophisticated.” “EP3 requires less effort for patients [understanding].” “Better navigation.” “Faster and easier.” “Simpler menus.”

^a^Participants were unaware about the functionality of the third generation of the easypod device, whereby, when an injection is performed, the screen is blocked, so that, if touched, nothing happens.

^b^HCP: health care professional.

^c^EP3: third generation of the easypod device.

^d^EP2: current easypod device.

### Simplified Touch Screen Interface

The development of a more intuitive interface that improved the clarity and visibility of information displayed and facilitated digital interaction was perceived as important by the participants:

The EP3 interface is more intuitive; easier to use [and] bigger visual clues.

Endocrinologist

Navigation is faster using touch screen than using buttons.

Nurse

Participants agreed that new-generation devices must include intuitive interfaces to ensure high usability. This fact was reflected on as part of the case study, in which participants agreed that the inclusion of a large touch screen in EP3 was a substantial improvement from EP2. They also confirmed that the interaction with the touch screen increased its ease of use and ease of learning; patients would be more familiar with this way of interaction because most of them are currently smartphone or tablet device users. In this regard, participants agreed that the use of EP3 is similar to using a smartphone. In addition, participants agreed that an intuitive interface is an important feature and consideration for future devices. For example, the simplifications included in the EP3 prototype (fewer steps to configure it or to access the appropriate option, and better navigation) were perceived as making it easier to use, learn, and train. This increased ease of learning and ease of training were considered as valuable by participants because making the device easy to teach and learn has a positive impact on HCPs’ clinical practice. HCPs, especially nurses, support patients and caregivers in the use of these digital devices. In this regard, HCPs introduce the device, explain its use, and resolve any questions from patients and caregivers; this requires time, which is quite limited in their daily practice.

### Real-Time Data Transmission

Participants repeatedly commented about the importance of collecting adherence data. They considered that data collection should be as transparent as possible for users, reducing the number of additional user interactions, and that the use of a digital solution is crucial for generating a sustainable, trusted, and unbiased adherence data collection method:

Measuring adherence is a crucial factor in any disease. The automatic data transmission allows to collect information that currently HCPs do not have access to.

Endocrinologist

Having real-time data collected automatically allows us to resolve doubts regarding adherence and improve the patient’s management.

Endocrinologist

Some HCPs reported their previous experience of using digital solutions in the management of pediatric chronic conditions, in which patients and caregivers did not share their adherence data because additional user actions required to transmit data were perceived as very burdensome. In this regard, the automatic process for real-time data transmission was considered to be a major advantage of the EP3 prototype, as it is transparent and independent of the user and does not require an additional device to transfer data, thus enabling HCPs’ access to real-time data from all patients. This was reported to be extremely valuable by HCPs because the decision-making process can be based on a more realistic data set than the one used previously. Participants, particularly nurses, also commented that training patients and caregivers on how to manage or self-manage pediatric disorders is a key factor, especially when using digital solutions. Participants agreed that the training process would be simplified because no instruction about how to transfer or share data would be required. Patient support programs have been developed to train patients and caregivers in the use of EP2 [17], but some of them do not acquire the appropriate skills or forget the process for transmission, leading to a lack of shared data. Thus, participants agreed that the automatic transmission of data would make the device easier to use, learn, and train. They also stated that features such as tactile interaction and automatic data transmission would both facilitate training and increase the usability of the device. In turn, this could reduce the time and effort required to train patients and caregivers.

Participants also agreed that the availability of real-time adherence data would enable better treatment monitoring and improved decision-making, as automatic data transmission offers a more reliable and realistic data set for both adherent and nonadherent patients, thus avoiding or reducing the current bias caused by the lack of data collected from poorly adherent patients.

### Administration Safety

Participants considered that administration problems such as false administration or unintended movements during treatment administration could be avoided by the EP3 prototype because of its large contact surface and the 360° skin sensor, which enables better skin contact than the 180° skin sensor in EP2:

The EP3 device presents improved processes [needle attachment, cartridge replacement, etc] making it easier to use.

Nurse

### Digital Ecosystem

Despite the advantages of the availability of real-time data, the participants acknowledged that the analysis of such data may increase their workload. As noted by the facilitators, all participants (10/10, 100%) agreed that the new device should be part of a digital health ecosystem that provides complementary functionalities such as data analysis and visualization:

I agree [that] new functionalities will be needed. These functionalities must automatically analyse the collected data and send an alarm/warning/alert to the HCP to be addressed.

Endocrinologist

Notifications and reminders with educational and motivational content as part of the overall digital health solution were seen as valuable additional elements.

### Additional Improvements

Relevant participants’ comments about the potential improvements of the case study have been included in this theme:

The EP3 [device] could be improved in terms of ergonomics, especially in [terms of] dimensions to be easily transported.

Nurse

The main area for improvement reported by participants related to the packaging of EP3; some found it difficult to open and close, adding that the size of packaging could make its transport and storage (in a refrigerator) difficult. Participants also commented that they had expected the EP3 prototype to be much lighter and smaller than EP2.

## Discussion

### Principal Findings

In the setting of pediatric GHD, the success of digital solutions—as part of integrated health care—requires an understanding of how HCPs perceive how connected devices facilitate patient management. This qualitative study, involving 10 HCPs from 7 reference hospitals in Spain, provides new information about the perceived usefulness and ease of use of a connected device that has evolved to meet the changing needs of those involved with the management of pediatric GHD. Participants in this study agreed that the new prototype device represents a technological evolution, in that it provides complementary functionalities—including real-time data analysis—and will require less time to explain and train both patients and caregivers in its use. Participants highlighted the inclusion of the large touch screen and real-time, automatic data transmission as the most relevant improvements. The automatic data transmission is transparent, with users having given consent and being aware that data will be transmitted to their HCP with no need for them to do anything to facilitate this. The functionality to automatically transmit data transparently contrasts with the results of a network analysis published in *The British Medical Journal* in 2019, which highlighted that sharing of user data from mobile apps is routine but far from transparent [51]. HCPs also agreed that access to real-time adherence data would enable better treatment monitoring, improved decision-making, and a more accurate evaluation of cost-effectiveness, which is consistent with observations by others [52]; this, in turn, has the potential to support and modify adherence behavior in patients receiving r-hGH treatment via the easypod device. Improved monitoring of adherence and availability of real-time data will enable more rapid intervention by HCPs and will ultimately improve outcomes, both in terms of growth outcomes and in reducing the long-term risks associated with GHD, including metabolic consequences.

The automatic transmission implemented in the new device will provide a more reliable and unbiased adherence data set. Data from both adherent and nonadherent patients would be collected, providing a more realistic scenario to evaluate adherence to treatment and, thus, the effectiveness of treatment on growth and other clinical outcomes, and orchestrating digital health interventions aimed at patients with low adherence. In the long term, it will also provide a more comprehensive national and global data set to support the development of more accurate prediction models and novel digital health interventions aimed at patients with low adherence [38]. However, some participants were concerned about the potential for increased workload because of the potentially large amount of collected data to be analyzed. This area requires further studies to determine the best approach for data analysis by HCPs, especially because the real-time data transmission feature of EP3 was considered as a major advantage by participants.

Participants also agreed that the digital device should ideally be a component of a connected digital ecosystem that provides complementary functionalities such as data analysis and visualization capabilities, delivery of alerts when any relevant event arose, and delivery of motivational messages. There is a need to create programs to support families and caregivers and connect them with their HCP for better management and understanding of the disease and to gain the full clinical benefits of the treatment, improve adherence, and reduce complications and related costs. This could be provided by means of an app downloaded to the patient’s or caregiver’s mobile phone. Such apps are already available or are in further development and refinement. A mobile app called growlink, a component of the easypod digital ecosystem, has been developed to be used by patients and their caregivers to monitor treatment progress and to receive relevant educational information to support them in their self-management of GHD, particularly as they transition from adolescence to adulthood [53]. Future developments of this app may include behavioral nudges, educational platforms, recording of patient-reported outcome measures, and programs providing psychological support; this, in turn, can promote positive changes in health behaviors and self-management of the condition [27,38,39].

Participants reported some negative opinions around ergonomics; size; and storage of EP3, particularly, storage in a refrigerator. In contrast, previous regulatory studies demonstrated that patients and caregivers were satisfied with the size of the device (unpublished data, Emergo by UL; unpublished data, Use-Lab). The increased height of EP3 was a result of a specific design decision to improve the accuracy of the dose administered; this resulted in a tall device but one with improved accuracy. However, ongoing studies to evaluate these factors from a user’s perspective will provide further valuable insights into these issues. Although some participants were concerned about the frontal location of the administration button, this was determined based on the results of human factor studies (unpublished data, Emergo by UL; unpublished data, Use-Lab). The participants’ comments about the need for small dose increments (depending on individual patient requirements based on clinical response and serum insulin-like growth factor–1 levels [54]) to be made available in the device settings were noted, and this is currently being investigated as part of the ongoing development of the EP3 prototype.

Our study presents an evaluation of connected injection devices to deliver r-hGH therapy using a robust methodological approach, the results of which are transferrable to digital health solutions in other therapeutic areas, especially in terms of facilitators for and barriers to technology acceptance. For example, a recent qualitative analysis concluded that barriers and facilitators should be considered for effective implementation of connected health solutions to support children with cancer and their families [35]. Although TAM is sometimes criticized for being very simplistic [55], the aim of our study was not to identify new constructs for TAM but to identify facilitators and barriers related to the core constructs of perceived usefulness and perceived ease of use that are common to other models and theories. These constructs are directly related to the technology being assessed and, therefore, are the most relevant factors for assessing how the technological advances could have an impact on HCP perspectives. However, the authors acknowledge that other frameworks can be used in this regard; for example, the Nonadoption, Abandonment, Scale-up, Spread, and Sustainability framework defines a construct directly related to technology that it is similar to our approach and is related to our findings [56]. Any future studies evaluating the acceptance of EP3 could use the Nonadoption, Abandonment, Scale-up, Spread, and Sustainability framework to explore other constructs such as “Value proposition” and “Adopters” [56].

### Study Limitations

A limitation of the study is the fact that it was conducted only in Spain, despite it providing a representative cross-sectional sample of HCPs who have used digital health solutions in clinical practice there. Exploring the perceptions of HCPs regarding EP3 in other countries and regions could be valuable to reflect views in other national and regional health care systems. Access to r-hGH treatment (from a practical and financial perspective) and advanced digital health solutions (including EP3) is likely to differ between countries. Finally, the small sample size does not allow the generalization of the quantitative data.

### Conclusions

This study explored the perceived value of the next-generation EP3 autoinjector device to HCPs, based on their assessment of the device to deliver r-hGH for the treatment of GHD compared with the currently used EP2. HCPs rated the new capabilities of the device, including the large touch screen and automatic data transmission, as substantial improvements. Therefore, this next-generation easypod device, while retaining the key features appreciated by patients such as the hidden needle and comfort settings, has the potential not only to improve and provide a more personalized treatment experience for patients and their caregivers but also to provide real-world and real-time insights for HCPs for improved clinical decision-making.

The overall conclusion of these participants was that the EP3 prototype was highly recommendable, based on their assessment from the viewpoint of HCPs involved in the treatment of growth disorders. It would be valuable to evaluate the perceptions about the usability of EP3 from the patient and caregiver perspective in future studies.

## Appendix

Multimedia Appendix 1 Items included in the Likert survey and mean Likert scores by domain.

## Abbreviations

EP2: current easypod device
EP3: third generation of the easypod device
GH: growth hormone
GHD: growth hormone deficiency
HBM: Health Belief Model
HCP: health care professional
HITAM: Health Information Technology Acceptance Model
ISO: International Organization for Standardization
mHealth: mobile health
r-hGH: recombinant human growth hormone
TAM: Technology Acceptance Model
TTF: Task-Technology Fit
UCD: user-centered design
UTAUT: Unified Theory of Acceptance and Use of Technology

## Data Availability

Any requests for data by qualified scientific and medical researchers for legitimate research purposes will be subject to the data sharing policy of the health care business of Merck KGaA, Darmstadt, Germany. All requests should be submitted in writing to the data sharing portal for the health care business of Merck KGaA, Darmstadt, Germany (https://tinyurl.com/5ary8j58). When the health care business of Merck KGaA, Darmstadt, Germany, has a coresearch, codevelopment, comarketing, or copromotion agreement or when the product has been out-licensed, the responsibility for disclosure might be dependent on the agreement between parties. Under these circumstances, the health care business of Merck KGaA, Darmstadt, Germany, will endeavor to gain agreement to share data in response to requests.
