# Supplementary material for: Evaluating the Usefulness and Ease of Use of a Next-Generation–Connected Drug Delivery Device for Growth Hormone Therapy: Qualitative Study of Health Care Professionals’ Perceptions
Source: JMIR Hum Factors. 2023 Aug 2;10:e46893. doi: 10.2196/46893 (PMC10433030; doi:10.2196/46893)
Supplement: Multimedia Appendix 1 [file humanfactors_v10i1e46893_app1.docx]

**Supplementary File**

**Evaluating the Usefulness and Ease of Use of a Next-Generation–Connected Drug Delivery Device for Growth Hormone Therapy: Qualitative Study of Health Care Professionals’ Perceptions**

**José I Labarta^1,2^, MD; Paul Dimitri^3^, BSc, MBChB; Matthew Keiser^4^, BS, MS; Ekaterina Koledova^5^, MD, PhD; Octavio Rivera-Romero^6^, PhD**

^1^Unit of Endocrinology, Department of Pediatrics, Hospital Universitario Miguel Servet, Zaragoza, Spain

^2^Instituto de Investigación Sanitaria Aragón, Zaragoza, Spain

^3^Department of Paediatric Endocrinology, Sheffield Children’s NHS Foundation Trust, Sheffield, United Kingdom

^4^Ares Trading SA (an affiliate of Merck KGaA), Eysins, Switzerland

^5^Global Medical Affairs Cardiometabolic & Endocrinology, Merck Healthcare KGaA, Darmstadt, Germany

^6^Electronic Technology Department*,* Universidad de Sevilla, Seville, Spain

**List of items included in the 25-item, 5-scale Likert survey**

| **Perceived usefulness** | |
| --- | --- |
| 1 | As an HCP, I consider that the functionalities included in the new device are useful for my work. |
| 2 | The new automatic data transmission process, that removes the need of use of an additional dock, is a significant advantage that will potentially impact on my work. |
| 3 | The new device allows HCPs to improve the care that they provide to patients. |
| 4 | The simplifications and improvements included in the new device allow HCPs to provide more effective care to the patient. |
| 5 | The new device allows its functionalities to be adapted to the individual’s needs. |
| 6 | The use of the new device will impact positively on patients’/caregivers’ self-management behaviours. |
| 7 | The use of the new device will impact positively on the patient’s/caregiver’s level of confidence increasing his/her knowledge and his/her level of understanding of the growth problem and its management. |
| 8 | The new improvements and simplifications included in the new device allow the patient/caregiver to administer the treatment in a safer way. |
| 9 | In general, as a professional, I think the new improvements included in the new device are positive. |
| **Perceived as easy to use** | |
| 10 | The dimensions and physical characteristics of the new prototype make the self-administration process easier for patients. |
| 11 | The dimensions and physical characteristics of the new prototype make the treatment administration process easier for caregivers. |
| 12 | The dimensions and physical characteristics of the new prototype allow for easier transportation. |
| 13 | The touch screen enables a more effective and comfortable interaction with the device. |
| 14 | The new device improves user interaction in terms of clarity and understanding (information and data are presented in a simpler and clear way). |
| 15 | The new automatic data transmission process simplifies patients’/caregivers’ tasks. |
| 16 | In general, the new improvements imply a reduced user effort to interact with the device. |
| 17 | In general, the improvements included in the new device simplify the treatment administration process (dose configuration, cartridge replacement, needle attachment/de-attachment, administration and data transmission) making it easier for users. |
| **Easy to learn** | |
| 18 | The new device simplifies tasks making its use easier to learn for HCPs. |
| 19 | The simplifications and improvements included in the new device make its use easier to learn for users. |
| 20 | The simplifications and improvements included in the new device make it easy to teach its use to the patient/caregiver for the HCP. |
| 21 | The simplifications and improvements included in the new device allow professionals to justify more easily the recommendation of its use for patients/caregivers. |
| **Intention to use and recommend** | |
| 22 | Patients will assess positively the improvements included in the new device. |
| 23 | As a professional, I consider my patients will be willing to use the new device. |
| 24 | I would recommend the use of the new device to my patients. |
| 25 | I would recommend the use of the new device to professionals who treat children with growth problems. |

HCP, healthcare professional.

**Supplementary Figure 1. Mean Likert scores by domain: Perceived usefulness, Perceived ease of use, Ease of learning and Intention to use and recommend**
